# Sodium–glucose cotransporter 2 inhibition does not improve the acute pressure natriuresis response in rats with type 1 diabetes

**DOI:** 10.1113/EP090849

**Published:** 2023-01-15

**Authors:** Natalie K. Jones, Hannah M. Costello, Marie‐Louise T. Monaghan, Kevin Stewart, David Binnie, Joanne Marks, Matthew A. Bailey, Geoffrey J. Culshaw

**Affiliations:** ^1^ British Heart Foundation Centre for Cardiovascular Science University of Edinburgh Edinburgh UK; ^2^ Department of Neuroscience Physiology and Pharmacology, Royal Free Campus University College London London UK

**Keywords:** blood pressure, diabetes, pressure natriuresis, SLGT2, sodium balance

## Abstract

Type 1 diabetes mellitus (T1DM) leads to serious complications including premature cardiovascular and kidney disease. Hypertension contributes importantly to these adverse outcomes. The renal pressure natriuresis (PN) response, a key regulator of blood pressure (BP), is impaired in rats with T1DM as tubular sodium reabsorption fails to down‐regulate with increasing BP. We hypothesised that sodium–glucose cotransporter 2 (SGLT2) inhibitors, which reduce cardiovascular risk in kidney disease, would augment the PN response in T1DM rats. Non‐diabetic or T1DM (35–50 mg/kg streptozotocin i.p.) adult male Sprague–Dawley rats were anaesthetised (thiopental 50 mg/kg i.p.) and randomised to receive either dapagliflozin (1 mg/kg i.v.) or vehicle. Baseline sodium excretion was measured and then BP was increased by sequential arterial ligations to induce the PN response. In non‐diabetic animals, the natriuretic and diuretic responses to increasing BP were not augmented by dapagliflozin. Dapagliflozin induced glycosuria, but this was not influenced by BP. In T1DM rats the PN response was impaired. Dapagliflozin again increased urinary glucose excretion but did not enhance PN. Inhibition of SGLT2 does not enhance the PN response in rats, either with or without T1DM. SGLT2 makes only a minor contribution to tubular sodium reabsorption and does not contribute to the impaired PN response in T1DM.

## Abstract (highlights)

- **What is the central question of this study?**
  Sodium–glucose cotransporter 2 (SGLT2) inhibitors reduce cardiovascular risk in patients with both diabetic and non‐diabetic kidney disease: can SGLT2 inhibition improve renal pressure natriuresis (PN), an important mechanism for long‐term blood pressure control, which is impaired in type 1 diabetes mellitus (T1DM)?
- **What is the main finding and its importance?**
  The SGLT2 inhibitor dapagliflozin did not enhance the acute in vivo PN response in either healthy or T1DM Sprague–Dawley rats. The data suggest that the mechanism underpinning the clinical benefits of SGLT2 inhibitors on health is unlikely to be due to an enhanced natriuretic response to increased blood pressure.

## 1 INTRODUCTION

The prevalence of type 1 diabetes mellitus (T1DM) in children and adolescents is around 1 in 300 in the USA (Maahs et al., 2010) and the incidence is increasing worldwide (Conway et al., 2012; Maahs et al., 2010). T1DM decreases life‐expectancy by ∼13 years (Livingstone et al., 2015), in part due to macrovascular and microvascular complications causing premature cardiovascular (CV) disease and nephropathy (Rawshani et al., 2017). Increased renal tubular sodium reabsorption (Song et al., 2003) and sodium retention (Feldt‐Rasmussen et al., 1987) are early hallmarks of clinical and experimental T1DM. Since renal regulation of extracellular fluid volume by modifying sodium reabsorption is a major determinant of long‐term blood pressure (BP) (Ivy & Bailey, 2014), dysfunction within the kidney impairs its ability to stabilise BP. Both hypertension and hyperglycaemia are major risk factors for CV disease and nephropathy (Conway et al., 2012; De Ferranti et al., 2014; Lee et al., 2019; Stehouwer & Smulders, 2006), and so restoration of the normal renal regulation of sodium balance is a therapeutic goal in order to reduce CV risk and further renal injury.

Pressure natriuresis (PN) is the positive relationship between BP, renal perfusion pressure and sodium excretion. This relationship, often attenuated in experimental hypertension, is hypothesised as a key regulator of long‐term BP (Guyton et al., 1972; Ivy & Bailey, 2014), although this remains controversial (Bie et al., 2004). Experimentally, PN largely reflects reduced sodium reabsorption in the proximal tubule due to inactivation of major sodium transport proteins (Ivy & Bailey, 2014). We have recently shown that the PN response is severely impaired in Sprague–Dawley rats with streptozotocin (STZ)‐induced T1DM (Culshaw et al., 2019). This dysfunction occurs because renal tubular sodium reabsorption does not down‐regulate following ramps in BP (Culshaw et al., 2019). The molecular basis of this impairment is not known.

Sodium–glucose cotransporter (SGLT)‐2 is the major route for glucose and sodium reabsorption in the proximal tubule, while SGLT1 in the S3 segment is responsible for a much smaller amount of reabsorption (Ghezzi et al., 2018). Acute inhibition of SGLT2 causes glycosuria, diuresis and natriuresis (Masuda et al., 2020; Zanchi et al., 2020). Chronically, SGLT2 inhibitors have been shown to reduce hyperglycaemia in patients with T1DM and type 2 diabetes mellitus (T2DM). They promote weight loss, prevent albuminuria and reduce BP (Fattah & Vallon, 2018; Tat & Forest, 2018), thus leading to a reduction in CV events and kidney failure in clinical trials (Groop et al., 2020; Neal et al., 2017; Perkovic et al., 2019). The reduction in CV and renal risk appears to be independent of the level of hyperglycaemia in patients (Perkovic et al., 2019), and following meta‐analysis of clinical trial data, the United States Food and Drug Administration (FDA) have recently approved the use of SGLT2 inhibitors in non‐diabetic patients with heart failure and reduced ejection fraction (Zannad et al., 2020). The mechanisms underpinning these clinical benefits remain elusive. Experimentally, SGLT2 inhibition is renoprotective, reducing markers of tubular and glomerular injury in the urine of diabetic rodent models (Jaikumkao et al., 2018; Oraby et al., 2019). Many anti‐hypertensive agents are able to stabilise extracellular fluid volume, reduce BP and protect glomeruli from an excessive hemodynamic load, and these effects are associated with an enhanced PN response (Ivy & Bailey, 2014; Saito & Kimura, 1996; Van Paassen et al., 2000). This raises the possibility that SGLT2 inhibitors enhance the acute PN response, and thereby reduce long‐term renal injury and CV risk.

We hypothesised that SGLT2 inhibition would restore the normal PN response in a rat model of T1DM. In this study, we recorded the natriuretic response after acute ramps in BP in anaesthetised healthy and T1DM rats and examined the effect of concurrent SGLT2 inhibition.

## 2 METHODS

### 2.1 Ethical approval

Experiments were performed in accordance with the UK's Animals (Scientific Procedures) Act under a UK Home Office Project License. All protocols were reviewed by the University of Edinburgh's Animal Welfare and Ethics Review Board prior to experimentation (Institutional Ethics Committee Approval No. 180‐LF2‐20).

### 2.2 Animals and husbandry

For all studies, adult male Sprague–Dawley rats (250–300 g) were purchased from Charles River (Tranent, UK) and were maintained on standard chow (0.25% sodium) and water ad libitum, and were housed in rooms with a 12‐h light cycle (lights 07.00−19.00 h) at 21 ± 1°C and 50% humidity.

### 2.3 Induction of T1DM

T1DM was induced by intraperitoneal (i.p.) injection of streptozotocin (STZ; 35 mg/kg in 0.1 M citrate buffer, pH 4.5; Sigma‐Aldrich, Gillingham, UK; *n* = 14). Rats had free access to food, and drinking water was supplemented with 10% sucrose to prevent initial hypoglycaemia. A blood glucose measurement 48 h later of >12 mmol/l on a glucometer (Accu‐Chek Aviva; Roche Diagnostics Ltd, Burgess Hill, UK; maximum blood glucose reading of 32 mmol/l) was required to confirm T1DM; rats that did not reach this threshold received a second injection of 15 mg/kg. Blood glucose was again measured at day 7 and immediately prior to the experimental procedure to confirm sustained hyperglycaemia. Non‐T1DM control rats (*n* = 16) received citrate vehicle alone by i.p. injection. All experiments were performed 2−3 weeks after the first STZ injection.

### 2.4 In vivo pressure natriuresis protocol

PN experiments were carried out as previously described (Culshaw et al., 2019) with all procedures beginning at around 10.00 h and rats having free access to food and drinking water until anaesthesia. Briefly, non‐recovery anaesthesia was induced with barbiturate anaesthetic agents. In the T1DM rats, sodium thiobutabarbital was used (Inactin; 120 mg/kg i.p.; Sigma‐Aldrich). Supply issues necessitated a change of anaesthetic, and for the non‐T1DM rats, sodium thiopental was used (50 mg/kg i.p.; Archimedes Pharma, Reading, UK). The right jugular vein was cannulated for intravenous (i.v.) infusion, a tracheotomy was performed and the right carotid artery was cannulated. The arterial line was used for intermittent blood sampling but otherwise was connected to a calibrated BP transducer and multi‐channel data acquisition system (Powerlab; ADInstruments, Oxford, UK) for real‐time mean arterial BP (MABP) measurement. Physiological saline (pH 7.4; 1 ml/h/100 g body weight), containing 2% bovine serum albumin and fluorescein isothiocyanate (FITC)–inulin (both Sigma‐Aldrich), was infused through the i.v. line. General anaesthesia was also maintained through this line by 20−30 μl injections of the barbiturate anaesthetic.

After a post‐surgical equilibration period of 30 min, the SGLT2 inhibitor dapagliflozin (1 mg/kg, Selleckchem, Munich, Germany, *n* = 7–8 per group) or vehicle (2% dimethyl sulfoxide in 0.9% saline, Sigma‐Aldrich, *n* = 7–8 per group) was injected through the i.v. line in a blinded fashion. After a 30 min baseline period, PN was induced by sequential arterial ligation of the celiac and cranial mesenteric arteries (period 1, 30 min), followed by a second ligation of the distal aorta (period 2, 30 min). During baseline and both periods of increased MABP, urine was collected via tube cystotomy for 30 min and the glomerular filtration rate (GFR) was measured as the ratio of urinary to plasma FITC–inulin concentrations multiplied by the urine flow rate per gram of kidney weight (kw). Rats were then culled by an overdose of anaesthetic followed by exsanguination via the carotid line.

Electrolyte analysis was carried out using a Spotchem EL SE‐1520 analyser (Arkray, Kyoto, Japan). Plasma and whole blood glucose were measured using the Accu‐Chek Aviva glucose meter (Roche). Glucose concentration in urine was measured by an enzymatic UV test using the hexokinase method (Beckman Coulter, High Wycombe, UK).

### 2.5 Statistical analysis

Statistical comparisons were made with GraphPad Prism 8 (GraphPad Software Inc., San Diego, CA, USA). Data are presented as individual data points with mean ± standard deviation (SD). The study was designed to obtain a power >80% if group sizes were six rats and dapagliflozin reduced the suppression of urinary sodium excretion during PN in T1DM rats by 50% (12 ± 6 μmol/min/g kw) (Culshaw et al., 2019). Additional rats were included in each group to account for anticipated dropouts. Overall, there was an experimental mortality rate of 6.25%; data and samples from these animals were not used in the subsequent analysis, resulting in final cohort sizes of *n* = 7 for T1DM rats and *n* = 8 for non‐T1DM rats. Because the anaesthetic agent had to be changed, comparisons were only made within groups (T1DM vs. T1DM, and non‐T1DM vs. non‐T1DM) to avoid potential confounding effects of the anaesthetic. Normality was assessed by the Shaprio–Wilk test with non‐normal data being log‐transformed prior to further analysis by two‐way analysis of variance (ANOVA). The fixed factors of BP ramps and dapagliflozin, and their interaction, generated three *P*‐values per comparison. Regression analysis plotted dependent variables from vehicle‐ and SGLT2 inhibitor‐treated rats against MABP (independent variable). An extra sum‐of‐squares *F*‐test was used to determine whether one curve fitted both data sets. For all tests, *P* < 0.05 was considered significant.

## 3 RESULTS

### 3.1 Pressure natriuresis and SGLT2 inhibition in non‐diabetic Sprague–Dawley rats

**FIGURE 1:**
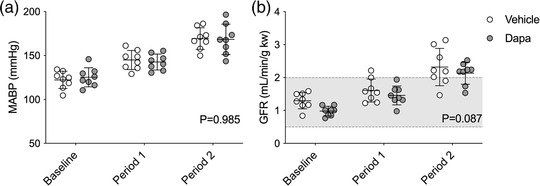
Changes in mean arterial blood pressure (MABP) and glomerular filtration rate (GFR) during the pressure natriuresis protocol. Sprague–Dawley rats were treated with a bolus i.v. injection of either vehicle (2% dimethyl sulfoxide in 0.9% saline, *n* = 8) or dapagliflozin (dapa, 1 mg/kg, *n* = 8) prior to stepwise arterial ligations of the celiac and cranial mesenteric arteries (period 1) and the distal aorta (period 2) to increase blood pressure under terminal anaesthesia. (a) MABP averaged over the 30‐min periods. (b) GFR measured using FITC–inulin. The shaded area represents the normal autoregulatory range of GFR in Sprague–Dawley rats (Culshaw et al., 2021). Individual data points and group mean ± standard deviation are shown. Statistical comparisons were made by 2‐way ANOVA where the main effect of dapagliflozin treatment is shown on each graph.

The PN response was measured in 16 male adult Sprague–Dawley rats. Eight rats received vehicle (weight, 399.6 ± 14.9 g; blood glucose, 7.5 ± 0.9 mM), and eight received dapagliflozin (weight, 397 ± 31.3 g; blood glucose, 7.4 ± 0.7 mM). MABP (Figure 1a) increased in both groups by a similar extent (effect of BP ramps, *P* < 0.0001; effect of dapagliflozin, *P* = 0.985; interaction, *P* = 0.629), with ramps of ∼20 mmHg (period 1) and ∼45 mmHg (period 2) from baseline. GFR values increased after ligation in a comparable manner (effect of BP ramps, *P* < 0.0001; effect of dapagliflozin, *P* = 0.087; interaction, *P* = 0.726), doubling from baseline in period 2 but still remaining within a range suggestive of effective autoregulation (Figure 1b).

**FIGURE 2:**
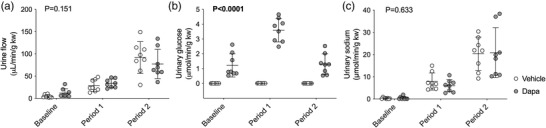
Changes in urine flow rate and urinary excretion of glucose and sodium. Urine flow rate (a), glucose (b) and sodium excretion (c) were measured in Sprague–Dawley rats treated with an i.v. bolus injection of either vehicle (2% dimethyl sulfoxide in 0.9% saline, *n* = 8) or dapagliflozin (dapa, 1 mg/kg, *n* = 8) prior to stepwise arterial ligations of the celiac and cranial mesenteric arteries (period 1) and the distal aorta (period 2) to increase blood pressure under terminal anaesthesia. Individual data points and group mean ± standard deviation are shown. Statistical comparisons were made by 2‐way ANOVA where the main effect of dapagliflozin treatment is shown on each graph.

From similar baseline urine flow rates (vehicle, 4.7 ± 3.4 μl/min/g kw; dapagliflozin, 12.1 ± 9.6 μl/min/g kw; *P* = 0.073), increases in BP induced a diuresis that was comparable between the two groups (effect of BP ramps, *P* < 0.0001; effect of dapagliflozin, *P* = 0.151; interaction, *P* = 0.001, Figure 2a). Dapagliflozin increased urinary glucose excretion at all time points (effect of BP ramps, *P* < 0.0001; effect of dapagliflozin, *P* < 0.0001; interaction, *P* = 0.071, Figure 2b). Urinary sodium excretion rates were also similar between groups at baseline (vehicle, 0.3 ± 0.3 μmol/min/g kw; dapagliflozin, 0.5 ± 0.7 μmol/min/g kw; *P* = 0.879) and both increased by ∼40‐fold when BP was increased (vehicle, 20.4 ± 7.5 μmol/min/g kw; dapagliflozin, 20.8 ± 11.2 μmol/min/g kw; effect of BP ramps, *P* < 0.0001; effect of dapagliflozin, *P* = 0.633; interaction, *P* = 0.848; Figure 2c).

**FIGURE 3:**
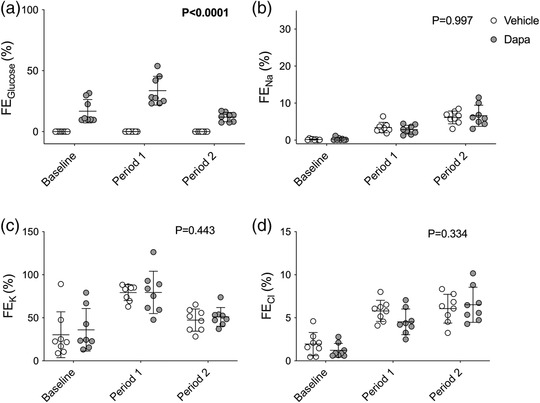
Fractional excretion (FE) of glucose, sodium, potassium and chloride. Urinary and plasma levels of glucose (a), sodium (b), potassium (c) and chloride (d) were measured in Sprague–Dawley rats treated with an i.v. bolus injection of either vehicle (2% dimethyl sulfoxide in 0.9% saline, *n* = 8) or dapagliflozin (dapa, 1 mg/kg, *n* = 8) prior to stepwise arterial ligations of the celiac and cranial mesenteric arteries (period 1) and the distal aorta (period 2) to increase blood pressure under terminal anaesthesia. The fraction of these molecules that was excreted rather than reabsorbed was calculated. Individual data points and group mean ± standard deviation are shown. Statistical comparisons were made by 2‐way ANOVA where the main effect of dapagliflozin treatment is shown on each graph.

The fractional excretions of sodium and glucose were then calculated to determine whether the natriuretic/glycosuric responses were due to a reduction in tubular reabsorption. As expected, dapagliflozin increased the fractional excretion of glucose but the magnitude varied according to the clearance period (effect of BP ramps, *P* < 0.0001; effect of dapagliflozin, *P* < 0.0001; interaction, *P* = 0.080; Figure 3a). Fractional excretion of sodium mirrored the increases in urine flow rates and sodium excretion rates with eightfold increases from baseline that were unaffected by dapagliflozin (effect of BP ramps, *P* < 0.0001; effect of dapagliflozin, *P* = 0.997; interaction, *P* = 0.667; Figure 3b). Similar results were also seen for potassium (effect of BP ramps, *P* = 0.0001; effect of dapagliflozin, *P* = 0.443; interaction, *P* = 0.685; Figure 3c) and chloride (effect of BP ramps, *P* < 0.0001; effect of dapagliflozin, *P* = 0.334; interaction, *P* = 0.181; Figure 3d).

**FIGURE 4:**
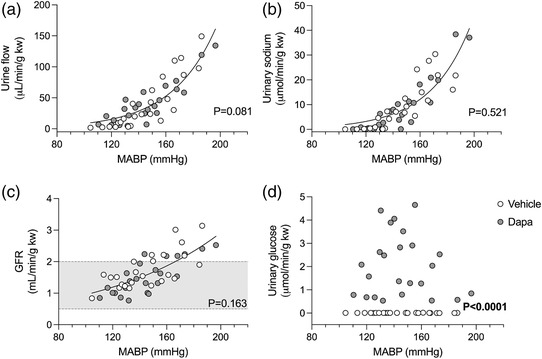
Pressure natriuresis response in Sprague–Dawley rats. Rats were treated with an i.v. bolus injection of either vehicle (2% dimethyl sulfoxide in 0.9% saline, *n* = 8) or dapagliflozin (dapa, 1 mg/kg, *n* = 8) prior to stepwise arterial ligations to increase mean arterial blood pressure (MABP) under terminal anaesthesia. MABP plotted against urine flow rate (a), urinary sodium excretion (b), urinary glucose excretion (c) and glomerular filtration rate (GFR) (d). Data analysed by a non‐linear fit curve with the null hypothesis of one curve fitting both data sets. Shaded area shows normal autoregulatory values for GFR in Sprague–Dawley rats (Culshaw et al., 2021). *P*‐values are shown on each graph.

The relationships of urine flow rate, urinary sodium excretion rate, and GFR with MABP were all curvilinear (Figure 4a–c). For each parameter, data sets from vehicle‐ and dapagliflozin‐treated rats could be fitted with a single curve (urine flow rate, *P* = 0.081; urinary sodium excretion, *P* = 0.521; GFR, *P* = 0.163). A regression line could not be fitted to either urinary glucose dataset, but all values from dapagliflozin‐treated rats were greater than those from vehicle‐treated rats (*P* < 0.0001, Figure 4d).

**FIGURE 5:**
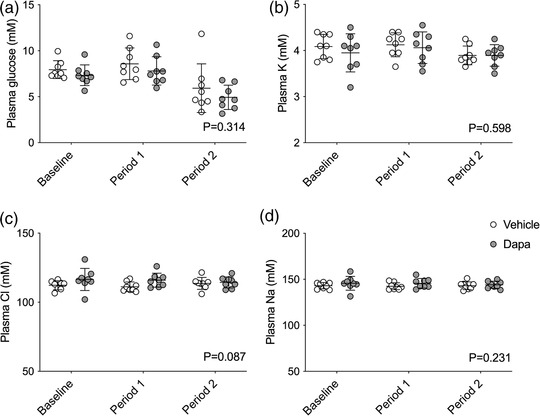
Plasma levels of glucose and electrolytes. Sprague–Dawley rats treated with an i.v. bolus injection of either vehicle (2% dimethyl sulfoxide in 0.9% saline, *n* = 8) or dapagliflozin (dapa, 1 mg/kg, *n* = 8) prior to stepwise arterial ligations of the celiac and cranial mesenteric arteries (period 1) and the distal aorta (period 2) to increase blood pressure under terminal anaesthesia. Glucose (a), sodium (b), chloride (c) and potassium (d) were measured in plasma at baseline and at each period. Individual data points and group mean ± standard deviation are shown. Statistical comparisons were made by 2‐way ANOVA where the main effect of dapagliflozin treatment is shown on each graph.

In contrast to the urine, plasma concentrations of glucose and electrolytes were relatively stable. When MABP increased, there were small reductions in plasma glucose by ∼2 mmol/l and potassium by ∼0.2 mmol/l, but these were unaffected by dapagliflozin (glucose: effect of BP ramps, *P* < 0.0001; effect of dapagliflozin, *P* = 0.314; interaction, *P* = 0.789; potassium: effect of BP ramps, *P* < 0.0001; effect of dapagliflozin, *P* = 0.598; interaction, *P* = 0.620; Figure 5a,b). Similarly, plasma chloride and sodium were unaffected by increases in MABP and did not differ between groups (chloride: effect of BP ramps, *P* = 0.778; effect of dapagliflozin, *P* = 0.087; interaction, *P* = 0.355; sodium: effect of BP ramps, *P* = 0.740; effect of dapagliflozin, *P* = 0.231; interaction, *P* = 0.715; Figure 5c,d).

### 3.2 Pressure natriuresis and SGLT2 inhibition in T1DM Sprague–Dawley rats

T1DM was induced by STZ injection in 14 Sprague–Dawley rats, and, 14 days later, they were randomly allocated to receive either vehicle (*n* = 7; weight, 376 ± 29 g; blood glucose, 29.6 ± 4.8 mmol/l) or dapagliflozin (*n* = 7; weight, 339 ± 33 g; blood glucose; 24.0 ± 7.7 mmol/l).

**FIGURE 6:**
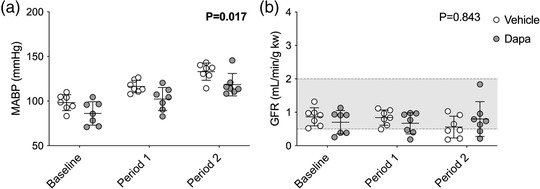
Mean arterial blood pressure (MABP) and glomerular filtration rate (GFR) during the pressure natriuresis protocol. Sprague–Dawley rats were treated with streptozotocin to induce type 1 diabetes and, 2 weeks later, were then treated with a bolus i.v. injection of either vehicle (2% dimethyl sulfoxide in 0.9% saline, *n* = 7) or dapagliflozin (dapa, 1 mg/kg, *n* = 7) prior to stepwise arterial ligations of the celiac and cranial mesenteric arteries (period 1) and the distal aorta (period 2) to increase blood pressure under terminal anaesthesia. (a) MABP averaged over the 30‐min periods. (b) GFR measured using FITC–inulin. The shaded area represents the normal autoregulatory range of GFR in Sprague–Dawley rats (Culshaw et al., 2021). Individual data points and group mean ± standard deviation are shown. Statistical comparisons were made by 2‐way ANOVA where the main effect of dapagliflozin treatment is shown on each graph.

MABP increased in both groups to a similar extent (effect of BP ramps, *P* < 0.0001; effect of dapagliflozin, *P* = 0.017; interaction, *P* = 0.889; Figure 6a), with ramps of ∼18 mmHg (period 1) and ∼35 mmHg (period 2) from baseline. Overall, MABP was ∼13 mmHg lower in dapagliflozin‐treated rats compared to vehicle but there were no differences between the groups during individual clearance periods. There was no difference in baseline GFR between groups and no measurable effect on GFR of increasing MABP (effect of BP ramps, *P* = 0.448; effect of dapagliflozin, *P* = 0.083; interaction, *P* = 0.054; Figure 6b).

**FIGURE 7:**
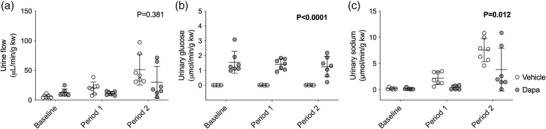
Urine flow and urinary excretion of glucose and sodium in type 1 diabetic rats. Urine flow rate (a), urinary glucose excretion (b) and sodium excretion (c) were measured in streptozotocin‐treated Sprague–Dawley rats then injected with an i.v. bolus of either vehicle (2% dimethyl sulfoxide in 0.9% saline, *n* = 7) or dapagliflozin (dapa, 1 mg/kg, *n* = 7) prior to stepwise arterial ligations of the celiac and cranial mesenteric arteries (period 1) and the distal aorta (period 2) to increase blood pressure under terminal anaesthesia. Individual data points and group mean ± standard deviation are shown. Statistical comparisons were made by 2‐way ANOVA where the main effect of dapagliflozin treatment is shown on each graph.

Increases in MABP induced a diuresis that was comparable between the dapagliflozin‐ and vehicle‐treated groups (effect of BP ramps, *P* < 0.0001; effect of dapagliflozin, *P* = 0.381; interaction, *P* = 0.001; Figure 7a) at either time point. Dapagliflozin treatment induced glycosuria, but vehicle did not, and there was no effect on glucose excretion by increasing MABP in either group (effect of BP ramps, *P* = 0.733; effect of dapagliflozin, *P* < 0.0001; interaction, *P* = 0.098; Figure 7b). Urinary sodium excretion rates were very similar between groups at baseline (vehicle, 0.2 ± 0.2 μmol/min/g kw; dapagliflozin, 0.2 ± 0.2 μmol/min/g kw; *P* = 0.383). Natriuresis was also induced by the BP ramps. However, despite increases in sodium excretion of up to ∼10‐fold (period 1) and ∼30‐fold (period 2) in both groups, the overall natriuretic effect was reduced by dapagliflozin (effect of BP ramps, *P* < 0.0001; effect of dapagliflozin, *P* = 0.012; interaction, *P* = 0.007; Figure 7c).

**FIGURE 8:**
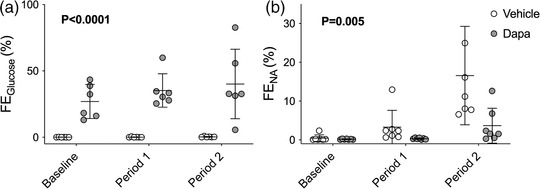
Fractional excretion (FE) of glucose and sodium. Urinary and plasma levels of glucose (a) and sodium (b) were measured in streptozotocin‐treated type 1 diabetic Sprague–Dawley rats treated with an i.v. bolus injection of either vehicle (2% dimethyl sulfoxide in 0.9% saline, *n* = 7) or dapagliflozin (dapa, 1 mg/kg, *n* = 7) prior to stepwise arterial ligations of the coeliac and cranial mesenteric arteries (period 1) and the distal aorta (period 2) to increase blood pressure under terminal anaesthesia to determine the fraction of these molecules that was excreted rather than reabsorbed. Individual data points and group mean ± standard deviation are shown. Statistical comparisons were made by 2‐way ANOVA where the main effect of dapagliflozin treatment is shown on each graph.

Fractional excretion of glucose was higher in the dapagliflozin‐treated group than vehicle‐treated controls throughout the protocol (effect of BP ramps, *P* = 0.012; effect of dapagliflozin, *P* < 0.0001; interaction, *P* = 0.019; Figure 8a). Fractional excretion of sodium was ∼0.3% at baseline in both vehicle‐ and dapagliflozin‐treated rats (Figure 8b). Acutely elevated BP increased fractional excretion of sodium in both groups, indicating a reduction in tubular sodium reabsorption, but the effect was blunted by dapagliflozin compared with vehicle (effect of BP ramps, *P* < 0.0001; effect of dapagliflozin, *P* = 0.005; interaction, *P* = 0.006).

**FIGURE 9:**
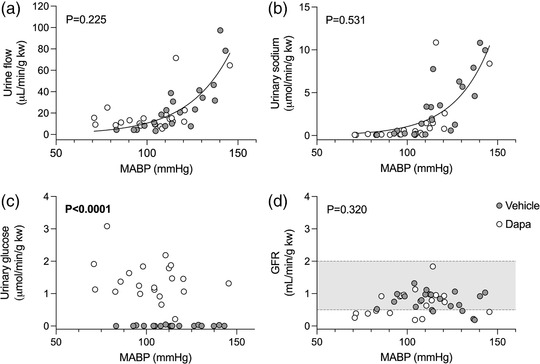
Pressure natriuresis responses in type 1 diabetic Sprague–Dawley rats. Diabetes was induced by streptozotocin injection 2 weeks prior to an i.v. bolus injection of either vehicle (2% dimethyl sulfoxide in 0.9% saline, *n* = 7) or dapagliflozin (dapa, 1 mg/kg, *n* = 7). Stepwise arterial ligations were carried out to increase mean arterial blood pressure (MABP) under terminal anaesthesia. MABP plotted against urine flow rate (a), urinary sodium excretion (b), urinary glucose excretion (c) and glomerular filtration rate (GFR) (d). Data analysed by a non‐linear fit curve with the null hypothesis of one curve fitting both data sets. Where curves could not be fitted, they were excluded from the graphs. Shaded area shows normal autoregulatory values for GFR in Sprague–Dawley rats (Culshaw et al., 2021). *P*‐values are shown on each graph.

The relationships of urine flow rate and urinary sodium excretion rate with MABP were curvilinear (Figure 9a,b). For both parameters, data sets from vehicle‐ and dapagliflozin‐treated rats could be fitted with a single curve (urine flow rate, *P* = 0.225; urinary sodium excretion, *P* = 0.531). A regression line could not be fitted to either GFR or urinary glucose datasets (Figure 9c,d). There was considerable overlap of dapagliflozin‐treated and vehicle‐treated GFR datasets.

## 4 DISCUSSION

The main finding of our study is that acute inhibition of sodium and glucose transport via SGLT2 with dapagliflozin does not enhance the experimental PN response. Of translational clinical importance, and contrary to our hypothesis, SGLT2 inhibition does not restore normal PN in T1DM rats. We conclude that SGLT2 makes only a minor contribution to overall tubular sodium reabsorption and does not contribute to the abnormal PN response in T1DM.

PN describes the renal response to acutely elevated BP; the rapid reduction in tubular sodium transport along the entire nephron can be measured as increased fractional excretion of sodium (Ivy & Bailey, 2014). In vivo, micropuncture confirms that due to autoregulation, the contribution that GFR makes to natriuresis is minor, whereas the largest contribution can be localised to inhibited sodium transport in the proximal tubule (Chou & Marsh, 1986, 1988). SGLT2 is also located within the proximal tubule, and when SGLT2 is inhibited and MABP maintained, glycosuria is accompanied by a ∼20% reduction in proximal tubular sodium reabsorption (Thomson et al., 2012). Therefore, we anticipated that our first PN experiment, in non‐T1DM rats, would demonstrate positive relationships not only between MABP and sodium excretion, but also MABP and glycosuria when SGLT2 was inhibited with dapagliflozin. Since we have previously identified an impaired PN response in pre‐nephropathy T1DM rats (Culshaw et al., 2019) that model sodium and water retention observed in people with T1DM (Feldt‐Rasmussen et al., 1987; O'Hare et al., 1989; Roland et al., 1986), we also used dapagliflozin in a separate PN experiment to try to restore these positive relationships in T1DM.

In both experiments, marked increases in MABP were achieved and increases in fractional excretion of sodium were consistent with the induction of PN. However, regardless of diabetic status, there was no positive relationship between MABP and urinary glucose, even during SGLT2 blockade. The absence of glycosuria in the T1DM rats receiving vehicle may have reflected a dip in blood glucose levels below the glycaemic threshold under anaesthesia, or increased SGLT2 activity. However, when SGLT2 was blocked, the impaired PN response we have observed in T1DM rats (Culshaw et al., 2019) still did not resolve. Therefore, overall sodium excretion and MABP are tightly linked, and this relationship is independent of sodium and glucose cotransport in the proximal tubule. In the context of the renal response to increased BP, the contribution of SGLT2‐mediated transport appears to be negligible.

The failure of blocking SGLT2 to enhance natriuresis in this PN model is worthy of further consideration because sodium and water retention contributes to cardiovascular risk in T1DM (Wenstedt et al., 2020). All rats treated with dapagliflozin had glycosuria, demonstrating that glucose reabsorption distal to SGLT2 is low, although previous studies have reported that SGLT1 activity increases in response to SGLT2 blockade (Lu et al., 2014). By contrast, overall sodium excretion did not increase, consistent with a compensatory increase in sodium transport within the nephron, downstream of SGLT2. Such a compensatory response is reported with other agents that interfere with sodium transport in the proximal tubule (Abdallah et al., 2001; Kaissling et al., 1985), but this has not been reported previously after acute SGLT2 blockade. Instead, increased rather than decreased urine output and sodium excretion have been described (Ansary et al., 2017). This discrepancy might be explained by our study design, in that, unlike previous studies, MABP was acutely increased rather than maintained at a baseline level. This should activate an integrated PN response that switches off sodium transporters along the nephron (Ivy & Bailey, 2014). An additional natriuretic response might not occur if any one of these transporters remained active after SGLT2 blockade. Identifying the transporter responsible was beyond the scope of this study and would require a combination of micropuncture and selective inhibition of sodium transport distal to the site of SGLT2 in the proximal tubule. However, compensatory reabsorption was also evident following dapagliflozin administration to T1DM rats, even when T1DM itself had already suppressed PN. Neither MABP nor GFR was reduced during individual clearance periods or on regression analysis, raising the possibility that reabsorption is driven by the sodium or glucose content of the tubular fluid or osmotic forces. This is already known to reduce the tubuloglomerular feedback signal in T1DM rats (Thomson & Vallon, 2021; Vallon et al., 2002), and therefore would be predicted to occur distal to the macula densa, where tubuloglomerular feedback is initiated.

The early T1DM model of this study was chosen because we are interested in the direct effect of SGLT2 blockade on renal sodium handling and the impaired PN response in T1DM. A more advanced T1DM model would introduce cofounding effects relating to nephropathy, such as renin–angiotensin–aldosterone system (RAAS) activation, and would not easily provide insight into the mechanism unpinning any changes to PN. We do not believe our results reflect RAAS activation because urinary aldosterone excretion is not increased in this pre‐nephropathy T1DM model (Culshaw et al., 2019), and in T1DM patients angiotensin II and plasma renin activity are reduced (Feldt‐Rasmussen et al., 1987; Thomsen & Shirley, 1997). Since disruption to BP regulation is thought to contribute to the development of nephropathy in T1DM (Lurbe et al., 2002), our study has implications for nephroprotection prior to nephropathy in T1DM. We also believe it may provide some explanation as to why sustained natriuresis or reductions in plasma volume are not observed in longer term clinical studies in people receiving SGLT2 inhibitors (Gal et al., 2020; Scholtes et al., 2021; Zanchi et al., 2020). Furthermore, since patients with T1DM already have difficulty in excreting an acute sodium load, our data do not support the use of SGLT2 inhibitors to improve this.

According to the Guyton hypothesis, sodium and water excretion are key determinants of BP (Guyton, 1987), so our data are also consistent with clinical trials that have demonstrated only a modest reduction in BP with SGLT2 inhibitors, despite inducing glycosuria (Kinaan et al., 2017), and a failure to promote natriuresis in T2DM patients over 2 weeks (Scholtes et al., 2021). However, care should be taken when placing our data within a clinical context. SGLT2 inhibitors do show promise in providing nephroprotection to T1DM patients (Groop et al., 2020) suggesting that even if SGLT2 blockade fails to improve natriuresis, the clinical consequences are not deleterious. We also did not specifically address the effects of chronic administration of an SGLT2 inhibitor on the acute PN response and therefore did not take into account the long‐term effects of SGLT2 blockade on the renal transcriptome, which can be profound, even inducing a phenotype that mimics fasting (Wu et al., 2022). At a functional level, in rats with congestive heart failure, where there is a drive to sodium and water retention, 4 weeks of empagliflozin enhanced excretion of an acute sodium load by downregulating proximal tubule NHE3‐activity (Borges‐Júnior et al., 2021), overwhelming any compensatory response downstream. This might be expected in people, since sustained dapagliflozin treatment enhances lithium clearance (Eickhoff et al., 2019), a marker of reduced proximal tubular sodium reabsorption, in T2DM. Therefore, to help determine the clinical implications from our work, PN experiments after long‐term administration of dapagliflozin are justified.

A further limitation of our study is that all the experimental rats used were exclusively male. Differences in the contribution of renal blood flow to PN are known to exist between male and female rats (Hilliard et al., 2011; Nakano & Pollock, 2009), and so extrapolation of our data to female rats should be made with caution. This is of clinical relevance since the CV benefits of SGLT2 inhibitors may be less in females than in males (Singh & Singh, 2020).

## AUTHOR CONTRIBUTIONS

All laboratory experiments were carried out at the British Heart Foundation Centre for Cardiovascular Science, University of Edinburgh, UK. Matthew A. Bailey and Geoffrey J. Culshaw designed the study and interpreted the findings, along with Joanne Marks. Natalie K. Jones, Hannah M. Costello, Marie‐Louise T. Monaghan, Kevin Stewart and David Binnie acquired and analysed the data gathered. Natalie K. Jones, Geoffrey J. Culshaw and Matthew A. Bailey drafted and revised the manuscript. All authors were involved in the critical evaluation of the manuscript for intellectual content, approved the final version of the manuscript, and agree to be accountable for all aspects of the work in ensuring that questions related to the accuracy or integrity of any part of the work are appropriately investigated and resolved. All persons designated as authors qualify for authorship, and all those who qualify for authorship are listed.

## CONFLICT OF INTEREST

The authors declare there are no conflicts of interest.

## Supporting information

Statistical Summary Document

## Data Availability

All supporting data are included within the main article or are available upon request by contacting the corresponding author.

## References

[eph13303-bib-0001] Abdallah, J. G. , Schrier, R. W. , Edelstein, C. , Jennings, S. D. , Wyse, B. , & Ellison, D. H. (2001). Loop diuretic infusion increases thiazide‐sensitive NA+/Cl—Cotransporter abundance: Role of aldosterone. Journal of the American Society of Nephrology, 12(7), 1335–1341. 11423562 10.1681/ASN.V1271335

[eph13303-bib-0002] Ansary, T. M. , Fujisawa, Y. , Rahman, A. , Nakano, D. , Hitomi, H. , Kobara, H. , Masaki, T. , Titze, J. M. , Kitada, K. , & Nishiyama, A. (2017). Responses of renal hemodynamics and tubular functions to acute sodium‐glucose cotransporter 2 inhibitor administration in non‐diabetic anesthetized rats /631/443/272/1684 /692/4022/272/1684 article. Scientific Reports, 7(1), 9555. 28842583 10.1038/s41598-017-09352-5 PMC5572725

[eph13303-bib-0003] Bie, P. , Wamberg, S. , & Kjolby, M. (2004). Volume natriuresis vs. Pressure natriuresis. Acta Physiologica Scandinavica, 181(4), 495–503. 15283763 10.1111/j.1365-201X.2004.01323.x

[eph13303-bib-0004] Borges‐Júnior, F. A. , Silva dos Santos, D. , Benetti, A. , Polidoro, J. Z. , Wisnivesky, A. C. T. , Crajoinas, R. O. , Antônio, E. L. , Jensen, L. , Caramelli, B. , Malnic, G. , Tucci, P. J. , & Girardi, A. C. C. (2021). Empagliflozin inhibits proximal tubule NHE3 activity, preserves GFR, and restores euvolemia in nondiabetic rats with induced heart failure. Journal of the American Society of Nephrology, 32(7), 1616–1629. 33846238 10.1681/ASN.2020071029 PMC8425656

[eph13303-bib-0005] Chou, C. L. , & Marsh, D. J. (1986). Role of proximal convoluted tubule in pressure diuresis in the rat. American Journal of Physiology. Renal Fluid and Electrolyte Physiology, 251(2), F283–F289. 10.1152/ajprenal.1986.251.2.F283 3740275

[eph13303-bib-0006] Chou, C. L. , & Marsh, D. J. (1988). Time course of proximal tubule response to acute arterial hypertension in the rat. American Journal of Physiology. Renal Fluid and Electrolyte Physiology, 254(4), F601–F607. 10.1152/ajprenal.1988.254.4.F601 3354689

[eph13303-bib-0007] Conway, B. R. , Rennie, J. , Bailey, M. A. , Dunbar, D. R. , Manning, J. R. , Bellamy, C. O. , Hughes, J. , & Mullins, J. J. (2012). Hyperglycemia and renin‐dependent hypertension synergize to model diabetic nephropathy. Journal of the American Society of Nephrology, 23(3), 405–411. 22193383 10.1681/ASN.2011060577 PMC3294297

[eph13303-bib-0008] Culshaw, G. , Binnie, D. , Dhaun, N. , Hadoke, P. , Bailey, M. , & Webb, D. J. (2021). The acute pressure natriuresis response is suppressed by selective ETA receptor blockade. Clinical Science, 136(1), 15–28. 34918049 10.1042/CS20210937 PMC8734438

[eph13303-bib-0009] Culshaw, G. J. , Costello, H. M. , Binnie, D. , Stewart, K. R. , Czopek, A. , Dhaun, N. , Hadoke, P. W. F. , Webb, D. J. , & Bailey, M. A. (2019). Impaired pressure natriuresis and non‐dipping blood pressure in rats with early type 1 diabetes mellitus. Journal of Physiology, 597(3), 767–780. 30537108 10.1113/JP277332 PMC6355628

[eph13303-bib-0010] De Ferranti, S. D. , De Boer, I. H. , Fonseca, V. , Fox, C. S. , Golden, S. H. , Lavie, C. J. , Magge, S. N. , Marx, N. , McGuire, D. K. , Orchard, T. J. , Zinman, B. , & Eckel, R. H. (2014). Type 1 diabetes mellitus and cardiovascular disease: A scientific statement from the American Heart Association and American Diabetes Association. Diabetes Care, 37(10), 2843–2863. 25114297 10.2337/dc14-1720 PMC4170130

[eph13303-bib-0011] Eickhoff, M. K. , Dekkers, C. C. J. , Kramers, B. J. , Laverman, G. D. , Frimodt‐Møller, M. , Jørgensen, N. R. , Faber, J. , Danser, A. H. J. , Gansevoort, R. T. , Rossing, P. , Persson, F. , & Heerspink, H. J. L. (2019). Effects of dapagliflozin on volume status when added to renin–angiotensin system inhibitors. Journal of Clinical Medicine, 8(6), 779. 31159350 10.3390/jcm8060779 PMC6616433

[eph13303-bib-0012] Fattah, H. , & Vallon, V. (2018). The potential role of SGLT2 inhibitors in the treatment of type 1 diabetes mellitus. Drugs, 78(7), 717–726. 29663292 10.1007/s40265-018-0901-y PMC6429906

[eph13303-bib-0013] Feldt‐Rasmussen, B. , Mathiesen, E. R. , Deckert, T. , Giese, J. , Christensen, N. J. , Bent‐Hansen, L. , & Nielsen, M. D. (1987). Central role for sodium in the pathogenesis of blood pressure changes independent of angiotensin, aldosterone and catecholamines in Type 1 (insulin‐dependent) diabetes mellitus. Diabetologia, 30(8), 610–617. 3653559 10.1007/BF00277316

[eph13303-bib-0014] Gal, A. , Burton, S. E. , Weidgraaf, K. , Singh, P. , Lopez‐Villalobos, N. , Jacob, A. , Malabu, U. , & Burchell, R. (2020). The effect of the sodium‐glucose cotransporter type‐2 inhibitor dapagliflozin on glomerular filtration rate in healthy cats. Domestic Animal Endocrinology, 70, 106376. 31585313 10.1016/j.domaniend.2019.07.004

[eph13303-bib-0015] Ghezzi, C. , Loo, D. D. F. , & Wright, E. M. (2018). Physiology of renal glucose handling via SGLT1, SGLT2 and GLUT2. Diabetologia, 61(10), 2087–2097. 30132032 10.1007/s00125-018-4656-5 PMC6133168

[eph13303-bib-0016] Groop, P. H. , Dandona, P. , Phillip, M. , Gillard, P. , Edelman, S. , Jendle, J. , Xu, J. , Scheerer, M. F. , Thoren, F. , Iqbal, N. , Repetto, E. , & Mathieu, C. (2020). Effect of dapagliflozin as an adjunct to insulin over 52 weeks in individuals with type 1 diabetes: Post‐hoc renal analysis of the DEPICT randomised controlled trials. The Lancet Diabetes and Endocrinology, 8(10), 845–854. 32946821 10.1016/S2213-8587(20)30280-1

[eph13303-bib-0017] Guyton, A. C. (1987). Renal function curve—A key to understanding the pathogenesis of hypertension. Hypertension, 10(1), 1–6. 3596763 10.1161/01.hyp.10.1.1

[eph13303-bib-0018] Guyton, A. C. , Coleman, T. G. , & Granger, H. J. (1972). Circulation: Overall regulation. Annual Review of Physiology, 34(1), 13–44. 10.1146/annurev.ph.34.030172.000305 4334846

[eph13303-bib-0019] Hilliard, L. M. , Nematbakhsh, M. , Kett, M. M. , Teichman, E. , Sampson, A. K. , Widdop, R. E. , Evans, R. G. , & Denton, K. M. (2011). Gender differences in pressure‐natriuresis and renal autoregulation: Role of the angiotensin type 2 receptor. Hypertension, 57(2), 275–282. 21189402 10.1161/HYPERTENSIONAHA.110.166827

[eph13303-bib-0020] Ivy, J. R. , & Bailey, M. A. (2014). Pressure natriuresis and the renal control of arterial blood pressure. Journal of Physiology, 592(18), 3955–3967. 25107929 10.1113/jphysiol.2014.271676 PMC4198007

[eph13303-bib-0021] Jaikumkao, K. , Pongchaidecha, A. , Chueakula, N. , Thongnak, L. O. , Wanchai, K. , Chatsudthipong, V. , Chattipakorn, N. , & Lungkaphin, A. (2018). Dapagliflozin, a sodium‐glucose co‐transporter‐2 inhibitor, slows the progression of renal complications through the suppression of renal inflammation, endoplasmic reticulum stress and apoptosis in prediabetic rats. Diabetes, Obesity and Metabolism, 20(11), 2617–2626. 10.1111/dom.13441 29923295

[eph13303-bib-0022] Kaissling, B. , Bachmann, S. , & Kriz, W. (1985). Structural adaptation of the distal convoluted tubule to prolonged furosemide treatment. American Journal of Physiology. Renal Fluid and Electrolyte Physiology, 248(3), F374–F381. 10.1152/ajprenal.1985.248.3.F374 3976898

[eph13303-bib-0023] Kinaan, M. , Yau, H. , Martinez, S. Q. , & Kar, P. (2017). Concepts in diabetic nephropathy: From pathophysiology to treatment. Journal of Renal and Hepatic Disorders, 1(2), 10–24.

[eph13303-bib-0024] Lee, Y. B. , Han, K. , Kim, B. , Lee, S. E. , Jun, J. E. , Ahn, J. , Kim, G. , Jin, S. M. , & Kim, J. H. (2019). Risk of early mortality and cardiovascular disease in type 1 diabetes: A comparison with type 2 diabetes, a nationwide study. Cardiovascular Diabetology, 18(1), 157. 31733656 10.1186/s12933-019-0953-7 PMC6858684

[eph13303-bib-0025] Livingstone, S. J. , Levin, D. , Looker, H. C. , Lindsay, R. S. , Wild, S. H. , Joss, N. , Leese, G. , Leslie, P. , McCrimmon, R. J. , Metcalfe, W. , McKnight, J. A. , Morris, A. D. , Pearson, D. W. M. , Petrie, J. R. , Philip, S. , Sattar, N. A. , Traynor, J. P. , & Colhoun, H. M. (2015). Estimated life expectancy in a scottish cohort with type 1 diabetes, 2008–2010. Journal of the American Medical Association, 313(1), 37–44. 25562264 10.1001/jama.2014.16425 PMC4426486

[eph13303-bib-0026] Lu, Y. , Griffen, S. C. , Boulton, D. W. , & Leil, T. A. (2014). Use of systems pharmacology modeling to elucidate the operating characteristics of SGLT1 and SGLT2 in renal glucose reabsorption in humans. Frontiers of Pharmacology, 5(10), 274. 10.3389/fphar.2014.00274 PMC4261707 25540623

[eph13303-bib-0027] Lurbe, E. , Redon, J. , Kesani, A. , Pascual, J. M. , Tacons, J. , Alvarez, V. , & Batlle, D. (2002). Increase in nocturnal blood pressure and progression to microalbuminuria in type 1 diabetes. New England Journal of Medicine, 347(11), 797–805. 12226150 10.1056/NEJMoa013410

[eph13303-bib-0028] Maahs, D. M. , West, N. A. , Lawrence, J. M. , & Mayer‐Davis, E. J. (2010). Epidemiology of type 1 diabetes. Endocrinology and Metabolism Clinics of North America, 39(3), 481–497. 20723815 10.1016/j.ecl.2010.05.011 PMC2925303

[eph13303-bib-0029] Masuda, T. , Muto, S. , Fukuda, K. , Watanabe, M. , Ohara, K. , Koepsell, H. , Vallon, V. , & Nagata, D. (2020). Osmotic diuresis by SGLT2 inhibition stimulates vasopressin‐induced water reabsorption to maintain body fluid volume. Physiological Reports, 8(2), e14360. 31994353 10.14814/phy2.14360 PMC6987478

[eph13303-bib-0030] Nakano, D. , & Pollock, D. M. (2009). Contribution of endothelin A receptors in endothelin 1‐dependent natriuresis in female rats. Hypertension, 53(2), 324–330. 19104001 10.1161/HYPERTENSIONAHA.108.123687 PMC2678242

[eph13303-bib-0031] Neal, B. , Perkovic, V. , Mahaffey, K. W. , de Zeeuw, D. , Fulcher, G. , Erondu, N. , Shaw, W. , Law, G. , Desai, M. , & Matthews, D. R. (2017). Canagliflozin and cardiovascular and renal events in Type 2 diabetes. New England Journal of Medicine, 377(7), 644–657. 28605608 10.1056/NEJMoa1611925

[eph13303-bib-0032] O'Hare, J. P. , Anderson, J. V. , Millar, N. D. , Dalton, N. , Tymms, D. J. , Bloom, S. R. , & Corrall, R. J. M. (1989). Hormonal response to blood volume expansion in diabetic subjects with and without autonomic neuropathy. Clinical Endocrinology, 30(5), 571–579. 2532573 10.1111/j.1365-2265.1989.tb01429.x

[eph13303-bib-0033] Oraby, M. A. , El‐Yamany, M. F. , Safar, M. M. , Assaf, N. , & Ghoneim, H. A. (2019). Dapagliflozin attenuates early markers of diabetic nephropathy in fructose‐streptozotocin‐induced diabetes in rats. Biomedicine and Pharmacotherapy, 109(1), 910–920. 30551545 10.1016/j.biopha.2018.10.100

[eph13303-bib-0034] Perkovic, V. , Jardine, M. J. , Neal, B. , Bompoint, S. , Heerspink, H. J. L. , Charytan, D. M. , Edwards, R. , Agarwal, R. , Bakris, G. , Bull, S. , Cannon, C. P. , Capuano, G. , Chu, P.‐L. , de Zeeuw, D. , Greene, T. , Levin, A. , Pollock, C. , Wheeler, D. C. , Yavin, Y. , … Mahaffey, K. W. (2019). Canagliflozin and renal outcomes in type 2 diabetes and nephropathy. New England Journal of Medicine, 380(24), 2295–2306. 30990260 10.1056/NEJMoa1811744

[eph13303-bib-0035] Rawshani, A. , Rawshani, A. , Franzén, S. , Eliasson, B. , Svensson, A. M. , Miftaraj, M. , McGuire, D. K. , Sattar, N. , Rosengren, A. , & Gudbjörnsdottir, S. (2017). Range of risk factor levels: Control, mortality, and cardiovascular outcomes in type 1 diabetes mellitus. Circulation, 135(16), 1522–1531. 28416524 10.1161/CIRCULATIONAHA.116.025961 PMC5400410

[eph13303-bib-0036] Roland, J. M. , O'Hare, J. P. , Walters, G. , & Corrall, R. J. M. (1986). Sodium retention in response to saline infusion in uncomplicated diabetes mellitus. Diabetes Research, 3(4), 213–215. 3527519

[eph13303-bib-0037] Saito, F. , & Kimura, G. (1996). Antihypertensive mechanism of diuretics based on pressure‐natriuresis relationship. Hypertension, 27(4), 914–918. 8613268 10.1161/01.hyp.27.4.914

[eph13303-bib-0038] Scholtes, R. A. , Muskiet, M. H. A. , Van Baar, M. J. B. , Hesp, A. C. , Greasley, P. J. , Karlsson, C. , Hammarstedt, A. , Arya, N. , Van Raalte, D. H. , & Heerspink, H. J. L. (2021). Natriuretic effect of two weeks of dapagliflozin treatment in patients with type 2 diabetes and preserved kidney function during standardized sodium intake: Results of the dapasalt trial. Diabetes Care, 44(2), 440–447. 33318125 10.2337/dc20-2604 PMC7818331

[eph13303-bib-0039] Singh, A. K. , & Singh, R. (2020). Gender difference in cardiovascular outcomes with SGLT‐2 inhibitors and GLP‐1 receptor agonist in type 2 diabetes: A systematic review and meta‐analysis of cardio‐vascular outcome trials. Diabetes and Metabolic Syndrome: Clinical Research and Reviews, 14(3), 181–187. 10.1016/j.dsx.2020.02.012 32142999

[eph13303-bib-0040] Song, J. , Knepper, M. A. , Verbalis, J. G. , & Ecelbarger, C. A. (2003). Increased renal ENaC subunit and sodium transporter abundances in streptozotocin‐induced type 1 diabetes. American Journal of Physiology. Renal Physiology, 285(6), F1125–F1137. 12904328 10.1152/ajprenal.00143.2003

[eph13303-bib-0041] Stehouwer, C. D. A. , & Smulders, Y. M. (2006). Microalbuminuria and risk for cardiovascular disease: Analysis of potential mechanisms. Journal of the American Society of Nephrology, 17(8), 2106–2111. 16825333 10.1681/ASN.2005121288

[eph13303-bib-0042] Tat, V. , & Forest, C. P. (2018). The role of SGLT2 inhibitors in managing type 2 diabetes. Journal of the American Academy of Physician Assistants, 31(6), 35–40. 10.1097/01.JAA.0000533660.86287.04 29846314

[eph13303-bib-0043] Thomsen, K. , & Shirley, D. G. (1997). The validity of lithium clearance as an index of sodium and water delivery from the proximal tubules. Nephron, 77(2), 125–138. 9346378 10.1159/000190264

[eph13303-bib-0044] Thomson, S. C. , Rieg, T. , Miracle, C. , Mansoury, H. , Whaley, J. , Vallon, V. , & Singh, P. (2012). Acute and chronic effects of SGLT2 blockade on glomerular and tubular function in the early diabetic rat. American Journal of Physiology. Regulatory Integrative and Comparative Physiology, 302(1), R75–R83. 21940401 10.1152/ajpregu.00357.2011 PMC3349378

[eph13303-bib-0045] Thomson, S. C. , & Vallon, V. (2021). Effects of SGLT2 inhibitor and dietary nacl on glomerular hemodynamics assessed by micropuncture in diabetic rats. American Journal of Physiology. Renal Physiology, 320(5), F761–F771. 33645318 10.1152/ajprenal.00552.2020 PMC8174804

[eph13303-bib-0046] Vallon, V. , Huang, D. Y. , Deng, A. , Richter, K. , Blantz, R. C. , & Thomson, S. (2002). Salt‐sensitivity of proximal reabsorption alters macula densa salt and explains the paradoxical effect of dietary salt on glomerular filtration rate in diabetes mellitus. Journal of the American Society of Nephrology, 13(7), 1865–1871. 12089382 10.1097/01.asn.0000016441.41118.57

[eph13303-bib-0047] Van Paassen, P. , De Zeeuw, D. , De Jong, P. E. , & Navis, G. (2000). Renin inhibition improves pressure natriuresis in essential hypertension. Journal of the American Society of Nephrology, 11(10), 1813–1818. 11004211 10.1681/ASN.V11101813

[eph13303-bib-0048] Wenstedt, E. F. E. , Rorije, N. M. G. , Olde Engberink, R. H. G. , Van Der Molen, K. M. , Chahid, Y. , Danser, A. H. J. , Van Den Born, B. J. H. , & Vogt, L. (2020). Effect of high‐salt diet on blood pressure and body fluid composition in patients with type 1 diabetes: Randomized controlled intervention trial. BMJ Open Diabetes Research and Care, 8(1), e001039. 10.1136/bmjdrc-2019-001039 PMC7228471 32404378

[eph13303-bib-0049] Wu, H. , Gonzalez Villalobos, R. , Yao, X. , Reilly, D. , Chen, T. , Rankin, M. , Myshkin, E. , Breyer, M. D. , & Humphreys, B. D. (2022). Mapping the single‐cell transcriptomic response of murine diabetic kidney disease to therapies. Cell Metabolism, 34(7), 1064–1078.e6. 35709763 10.1016/j.cmet.2022.05.010 PMC9262852

[eph13303-bib-0050] Zanchi, A. , Burnier, M. , Muller, M. E. , Ghajarzadeh‐Wurzner, A. , Maillard, M. , Loncle, N. , Milani, B. , Dufour, N. , Bonny, O. , & Pruijm, M. (2020). Acute and chronic effects of SGLT2 inhibitor empagliflozin on renal oxygenation and blood pressure control in nondiabetic normotensive subjects: A randomized, placebo‐controlled trial. Journal of the American Heart Association, 9(13), e016173. 32567439 10.1161/JAHA.119.016173 PMC7670540

[eph13303-bib-0051] Zannad, F. , Ferreira, J. P. , Pocock, S. J. , Anker, S. D. , Butler, J. , Filippatos, G. , Brueckmann, M. , Ofstad, A. P. , Pfarr, E. , Jamal, W. , & Packer, M. (2020). SGLT2 inhibitors in patients with heart failure with reduced ejection fraction: A meta‐analysis of the EMPEROR‐Reduced and DAPA‐HF trials. The Lancet, 396(10254), 819–829. 10.1016/S0140-6736(20)31824-9 32877652

